# New diagonal micropolarizer arrays designed by an improved model in fourier domain

**DOI:** 10.1038/s41598-021-85103-x

**Published:** 2021-03-11

**Authors:** Jia Hao, Yan Wang, Kui Zhou, Xiaochang Yu, Yiting Yu

**Affiliations:** 1 Research & Development Institute of Northwestern Polytechnical University in Shenzhen, Shenzhen, 518057 China; 2 Key Laboratory of Micro/Nano Systems for Aerospace (Ministry of Education), Northwestern Polytechnical University, Xi’an, 710072 China; 3 Shaanxi Province Key Laboratory of Micro and Nano Electro-Mechanical Systems, Northwestern Polytechnical University, Xi’an, 710072 China

**Keywords:** Imaging and sensing, Optical sensors, Optoelectronic devices and components

## Abstract

The design of micropolarizer array (MPA) patterns in Fourier domain provides an efficient approach to reconstruct and investigate the polarization information. Inspired by Alenin’s works, in this paper, we propose an improved design model to cover both 2 × N MPAs and other original MPAs, by which an entirely new class of MPA patterns is suggested. The performance of the new patterns is evaluated through Fourier domain analysis and numerical simulations compared with the existing MPAs. Particularly, we analyze the reconstruction accuracy of the first three Stokes parameters and degree of linear polarization (DoLP) in detail. The experimental results confirm that the 2 × 2 × 2 MPA provides the highest reconstruction quality of *s*_0_, *s*_1_, *s*_2_ and DoLP in terms of quantitative measures and visual quality, while the 3 × 3 diagonal MPA achieves the state-of-the-art best results in case of single-snapshot systems. The guidance of this extended model and new diagonal MPAs show its massive potential for the division of focal plane (DoFP) polarization imaging applications.

## Introduction

At present, the most attractive polarization imaging instrument employs the way of division of focal plane (DoFP), in which a micropolarizer array (MPA) is placed in front of the camera’s focal plane, showing the advantages of simple structure, compact size and the capability of real-time detection^1,2^. The first polarization imaging system which had been built using the conventional 2 × 2 repeating pattern of polarization analyzer, was introduced by Chun^3^ in 1994 and has been a standard for almost 20 years. However, since each pixel within a 2 × 2 minimum periodic array can only collect the intensity information of its corresponding polarization orientation^4^, the spatial resolution is reduced, and the reconstructed polarization information is not accurate due to the instantaneous field of views (IFOV)^5,6^. Therefore, it is significant to enhance the quality of polarization reconstruction images for further applications.

Until now, various methods have been developed for the quality enhancement of polarization reconstruction images. Subsequently, several works have demonstrated that both the layout of MPAs and the reconstruction algorithms affect the quality of the reconstructed Stokes parameter images^7–11^. Furthermore, some unconventional MPA patterns^7,8^ and interpolation methods^6,9–11^ have been proposed to achieve superior resolution and accurate polarization information. For example, in 2014, LeMaster and Hirakawa^7^ creatively put forward a novel 2 × 4 MPA pattern, which demonstrated that the high reconstruction quality could be achieved over the traditional 2 × 2 MPA. For further exploration, in 2017, Alenin et al.^8^ designed 2 × 3, 2 × 7 and 2 × 2 × 2 MPA patterns for linear DoFP polarimeters, and extended the 2 × 4 MPA into a family of 2 × N MPAs aiming to widen the optimization space in Frequency Domain. The results indicated that the maximized theoretical spatial resolution of the reconstructed Stokes parameters could be achieved by the 2 × 2 × N MPAs. Despite their previous superior efforts advanced the available set of MPA patterns and improved the reconstruction quality of Stokes parameters and degree of linear polarization (DoLP) images, however, these works were merely focused on the family of 2 × N patterns, and although the 2 × 2 × N MPAs have superior performance in reconstruction quality, these MPAs should be used in multiple-snapshots systems. Besides, to the best of our knowledge, the application of a generalized model to investigate new MPA layouts for improving reconstruction accuracy has not been reported. Therefore, for the purpose of a higher reconstruction quality in case of single-snapshot systems, designing higher-performance MPA patterns for linear DoFP polarimeters is required.

Motivated by the extraordinary design model^8^, in this paper, we propose an improved model that can be leveraged to design both the 2 × N MPA patterns and N × N diagonal MPA patterns by changing the location of carriers in Fourier domain. Moreover, to compare the performance of those previously demonstrated MPAs with the entirely new class of N × N diagonal MPAs, we provide a detailed comparison of the reconstruction quality in *s*_0_, *s*_1_, *s*_2_ and DoLP simultaneously. The results demonstrate that this improved model can further expand the scope of MPAs and effectively design a new type of patterns with higher performance, which may bring a great applications for the DoFP polarization detection technique.

## Methodology and design

### Design model of MPAs

Stokes vector **S** has been proven to be efficient for representing the polarization states of incident light^1^. For DoFP systems, most digital cameras combine a photodetector with a MPA to capture the incident light. Then the Stokes vector can be indirectly calculated using the acquired intensity images from different polarization orientations. Traditionally, it is formulated as:

1 $${\mathbf{S}} = \left[ {\begin{array}{*{20}l} {s_{0} } \hfill \\ {s_{1} } \hfill \\ {s_{2} } \hfill \\ {s_{3} } \hfill \\ \end{array} } \right] = \left[ {\begin{array}{*{20}l} {{{\left( {I_{{0^{^\circ } }} + I_{{45^{^\circ } }} + I_{{90^{^\circ } }} + I_{{135^{^\circ } }} } \right)} \mathord{\left/ {\vphantom {{\left( {I_{{0^{^\circ } }} + I_{{45^{^\circ } }} + I_{{90^{^\circ } }} + I_{{135^{^\circ } }} } \right)} 2}} \right. \kern-\nulldelimiterspace} 2}} \hfill \\ {I_{{0^{^\circ } }} - I_{{90^{^\circ } }} } \hfill \\ {I_{{45^{^\circ } }} - I_{{135^{^\circ } }} } \hfill \\ {I_{R} - I_{L} } \hfill \\ \end{array} } \right],$$

where *I*_0°_, *I*_45°_, *I*_90°_, and *I*_135°_ represent the intensities measured through a linear polarizer in the directions of 0°, 45°, 90°, and 135°, respectively, and *s*_3_ is always ignored for linear DoFP polarimeters^13^.

**Figure 1:**
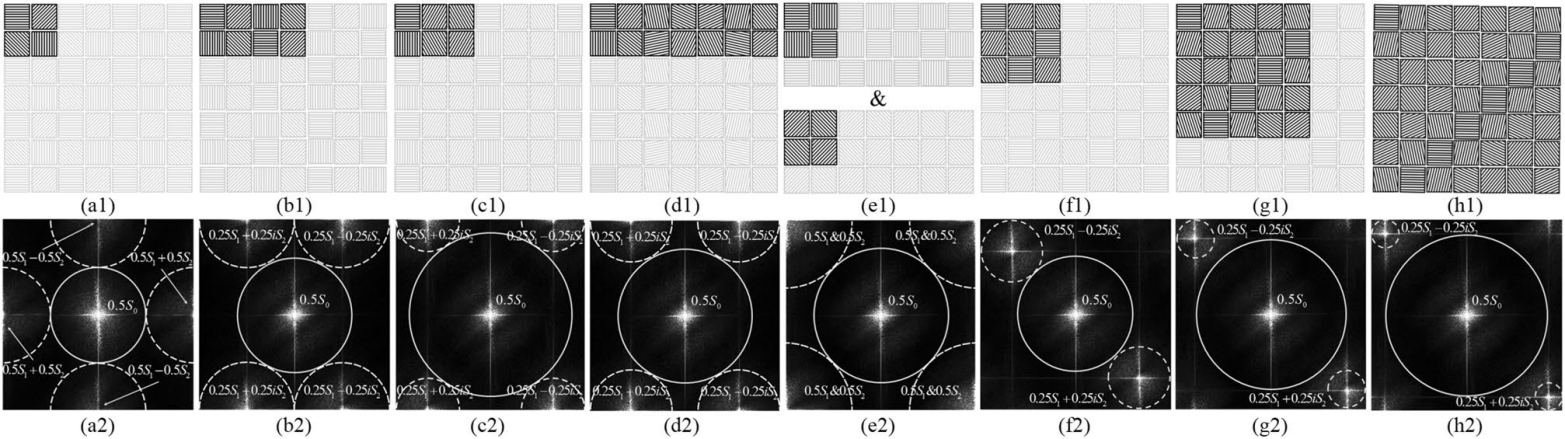
Eight MPA patterns and their corresponding Discrete Fourier Transform (DFT) spectra. The first row represents the (**a1**) conventional 2 × 2^3^ (**b1**) LeMaster’s 2 × 4^7^, (**c1**) Alenin’s 2 × 3^8^, (**d1**) Alenin’s 2 × 7^8^, (**e1**) Alenin’s 2 × 2 × 2^8^, (**f1**) this work’s 3 × 3, (**g1**) this work’s 5 × 5, (**h1**) this work’s 7 × 7. The second row gives the spectra according to MPAs in the first row.

To derive Stokes parameters, the captured image **I** is given using a series of pre-determined analyzing polarization states, **A**_N_^8^,

2 $$\begin{aligned} {\mathbf{I}} & = \left[ {\begin{array}{*{20}l} {I_{1} } \hfill & \cdots \hfill & {I_{N} } \hfill \\ \end{array} } \right]^{T} = \left[ {\begin{array}{*{20}l} {{\mathbf{\rm A}}_{{\mathbf{1}}}^{{\mathbf{\rm T}}} {\mathbf{S}}} \hfill & \cdots \hfill & {{\mathbf{A}}_{{\mathbf{N}}}^{{\mathbf{T}}} {\mathbf{S}}} \hfill \\ \end{array} } \right]^{T} + {\varvec{n}} \\ & = {\mathbf{WS}} + {\varvec{n}}, \\ \end{aligned}$$

where I_N_ represents the intensity of the polarized light passing through the linear polarizer, **S** represents the unknown polarization state, and ***n*** represents an additive detector noise. Then the unknown state’s Stokes parameters can be revealed by,

3 $$\widehat{{\mathbf{S}}} = {\mathbf{W}}^{{\mathbf{ + }}} {\mathbf{I}} = {\mathbf{W}}^{{\mathbf{ + }}} {\mathbf{WS}} + {\mathbf{W}}^{{\mathbf{ + }}} \user2{n },$$

where $\widehat{{\mathbf{S}}}$ represents the calculated Stokes parameters and “+” in the upper right corner denotes the pseudo-inverse of a matrix. Because the layout of the MPA is periodic and defined by the square lattice, it is convenient to design polarization orientations of MPAs using an analysis vector. The conventional and most commonly used 2 × 2 MPA, as shown in Fig. 1a1, and the available set of 2 × N MPAs, as shown in Fig. 1b1–e1 can be expressed by using the analyzing vector^8^,

4 $${\mathbf{A}}_{m,n} = \frac{1}{4}\left[ {\begin{array}{*{20}l} 2 \hfill \\ {\cos \left( {m\pi } \right) + \cos \left( {n\pi } \right)} \hfill \\ {\cos \left( {m\pi } \right) - \cos \left( {n\pi } \right)} \hfill \\ \end{array} } \right],\;{\text{or}}\;{\mathbf{A}}_{m,n} = \frac{1}{2}\left[ {\begin{array}{*{20}l} 1 \hfill \\ {\cos \left( {am\pi } \right)\cos \left( {bn\pi } \right)} \hfill \\ {\sin \left( {am\pi } \right)\cos \left( {bn\pi } \right)} \hfill \\ \end{array} } \right],$$

where *m* and *n* are the horizontal and vertical pixel coordinates in a MPA pattern, respectively. The polarization orientations of each pixel can be calculated by changing the *m* and *n*, while *a* and *b* determine the positioning of carriers in horizontal and vertical direction, respectively.

Inspired by the above design model, we propose a new generalized model, which breaks the limit of Eq. (4) and can be used to design a novel type of MPAs with N × N diagonal configuration. The permutation of three N × N diagonal MPAs, as s hown in Fig. 1e1–g1, belong to the following analyzing vector,

5 $${\mathbf{A}}_{m,n} = \frac{1}{2}\left[ {\begin{array}{*{20}l} 1 \hfill \\ {\cos \left[ {\frac{2\pi }{a}(bm + cn)} \right]} \hfill \\ {\sin \left[ {\frac{2\pi }{a}(bm + cn)} \right]} \hfill \\ \end{array} } \right],$$

by which, both the LeMaster’s 2 × 4 MPA and Alenin’s 2 × N MPAs can also be designed by setting the appropriate parameters. As shown in Table 1, the corresponding parameters of the 2 × N MPAs and the N × N diagonal MPAs are discussed in greater detail.

**Table 1:** Notable micropolarizer array patterns.

Type	Origin	Size	*a*	*b*	*c*	N	CN^a^	$\sigma_{0,1,2,3}^{2}$^b^	Q^T^	Q^+^
2 × N	Chun ^3^	2 × 2	–	–	–	1	$\sqrt 2$	$1,4,4,\Phi$		
	LeMaster ^7^	2 × 4	4	2	1	1	$\sqrt 2$	$1,4,4,\Phi$		
	Alenin ^8^	2 × 3	3	1.5	1	1	$\sqrt 2$	$1,4,4,\Phi$		
	Alenin ^8^	2 × 7	7	3.5	2	1	$\sqrt 2$	$1,4,4,\Phi$		
	Alenin ^8^	2 × 2 × 2	2	1	1	2	$\sqrt 2$	$\frac{1}{2},4,4,\Phi$		
Diag N × N	This work	3 × 3	3	1	1	1	$\sqrt 2$	$1,4,4,\Phi$		
	This work	5 × 5	5	2	2	1	$\sqrt 2$	$1,4,4,\Phi$		
	This work	7 × 7	7	3	3	1	$\sqrt 2$	$1,4,4,\Phi$		

The circles represent the coefficient’s polar form of the frequency phase matrix, and the total number of circles is 9 × 4 = 36. The direction of the radius line in the circles represents the phase information as: right =  + 1, up =  + j, left =  − 1, down =  − j. The frequency phase matrix in Eq. (7) indicates that there have nine channels, which contain four empty channels and five non-empty channels. Empty circles indicate that the locations of the channels do not contain the information of Stokes parameter^8^.

^a^CN is the condition number, which ensures the optimal configuration for **W**^12^.

^b^σ^2^_0,1,2,3_ are the noise variances from **Q**, which is a 9 × 4 matrix; the EWV is the sum of σ^2^_0,1,2,3_.

The intensity measured through linear polarizer with different directions can be represented by,

6 $$I_{\theta } = (s_{0} + s_{1} \cos 2\theta + s_{2} \sin 2\theta )/2,$$

where *θ* represents the orientation angle of analyzer at different location. According to the Eqs. (5) and (6), it is convenient to calculate the orientation angles of the MPAs designed by our proposed model.

### MPA’s performance evaluations

As shown in Alenin’s seminal work^8^, the Fourier transform of Eq. (5) can be denoted by the Frequency Phase Matrix (FPM) to describe the corresponding channel structure, which provides new insights into MPA patterns design,

7 $${\hat{\mathbf{A}}}_{\xi ,\eta } = \left[ {\begin{array}{*{20}l} {\frac{1}{2}\delta (\xi ,\eta )} \hfill \\ {\frac{1}{4}\left[ {\delta \left( {\xi + \frac{b}{a},\eta + \frac{c}{a}} \right) + \delta \left( {\xi - \frac{b}{a},\eta - \frac{c}{a}} \right)} \right]} \hfill \\ {\frac{j}{4}\left[ {\delta \left( {\xi + \frac{b}{a},\eta + \frac{c}{a}} \right) - \delta \left( {\xi - \frac{b}{a},\eta - \frac{c}{a}} \right)} \right]} \hfill \\ \end{array} } \right],$$

where *a*, *b* and *c* determine the positioning of carriers in Fourier domain.

For the purpose of analyzing the proposed model theoretically, we use the **Q** formalism to analyze the Stokes polarimeters, which can reveal the relationship between the Stokes parameters and the set of channel structures^8,13^. By properly inverting process, the Stokes vector can also be reconstructed by,

8 $$\widehat{{\mathbf{S}}} = F^{ - 1} \left\{ {{\mathbf{Q}}^{{\mathbf{ + }}} F\left\{ {\mathbf{C}} \right\}} \right\},$$

where **Q**^**+**^ is the pseudo-inverse of **Q**, and **C** is the related channel. Accordingly, the parameters of MPAs designed by Eq. (5), the corresponding channel structures indicated by the **Q** matrix and its inverse are all shown in Table 1.

Based on the Alenin’s theory^14^, to assess the performance of a MPA while taking the noise, bandwidth, and system error into consideration, using the equally weighted variance (EWV) inferred by **Q**^**+**^ as an index is significant. It provides a handy way to evaluate the intrinsic properties of the channel structure. As shown in Table 1, the family of N × N diagonal MPAs has the same value of EWV as the family of 2 × N MPAs. In particular, we can also design the 2 × 2 × 2 MPA with the setting of *a* = 2 and *b* = *c* = 1 in our proposed model, whose EWV is equal to 8.5. Although this type of MPA extends the channel centers to the outer corners and provides a strong capability of noise resilience, this MPA cannot reconstruct the first three Stokes parameters in real-time^8^. To obtain all the mentioned Stokes parameters simultaneously, it must add a temporally modulated ferroelectric liquid crystal rotator. As an appropriate metric, the concept of EWV can be used to evaluate the performance and assess noise immunity. However, the bandwidth also has an effect on the quality of reconstruction, which improves when the higher frequency information is included. Therefore, the design of MPA patterns should take into account various factors comprehensively, and the effective analysis of the bandwidth of *s*_0_, *s*_1_ and *s*_2_ is significant for evaluating the MPA’s performance.

As shown in Fig. 1, it is noticeable that the spectra have several multiplex frequency components different by the location and the bandwidth in Fourier domain. White solid lines outline the channel bandwidths of *s*_0_, and white dotted lines outline the channel bandwidths of multiplex components of *s*_1_ and *s*_2_. Note that by convention, the basebands are placed at the center in the second row, but all the DFT spectra are periodic in both horizontal and vertical directions. Based on the Fourier domain representation, it is intuitive and visual to analyze the location of multiplex frequency components, the extent of aliasing contaminations and relative bandwidths between different components. As shown in Fig. 1a2, we can easily discover that the conventional 2 × 2 MPA has a small theoretical bandwidth of *s*_0_, because the frequency components overlap on the horizontal and vertical axes. Obviously, as shown in Fig. 1b2–h2, the 2 × N MPAs and N × N diagonal MPAs remarkably reduce the risk of aliasing and improve the bandwidth of *s*_0_ by further separating *s*_1_ and *s*_2_ carriers away from the *s*_0_ carrier. Therefore, these MPAs can significantly reduce the crosstalk impact of different components and improve the reconstruction quality as the theoretical bandwidth of *s*_0_ increases. Similarly, we can analyze the theoretical bandwidth of *s*_1_ and *s*_2_ with the same approach. Avoiding the frequency components situated on the horizontal and vertical axes, the reconstruction accuracy of *s*_1_ and *s*_2_ increases strikingly as the theoretical bandwidth increases. Based on these analyses, we can see that the 2 × 2 × 2 MPA provides the best *s*_0_, *s*_1_ and *s*_2_ reconstruction accuracy in case of multiple-snapshots systems, while the 7 × 7 diagonal MPA provides the best *s*_0_ reconstruction accuracy in case of single-snapshot systems theoretically. Furthermore, the quality of the DoLP should be weighed depending on various factors comprehensively.

## Experimental validation

### Reconstruction method

**Figure 2:**
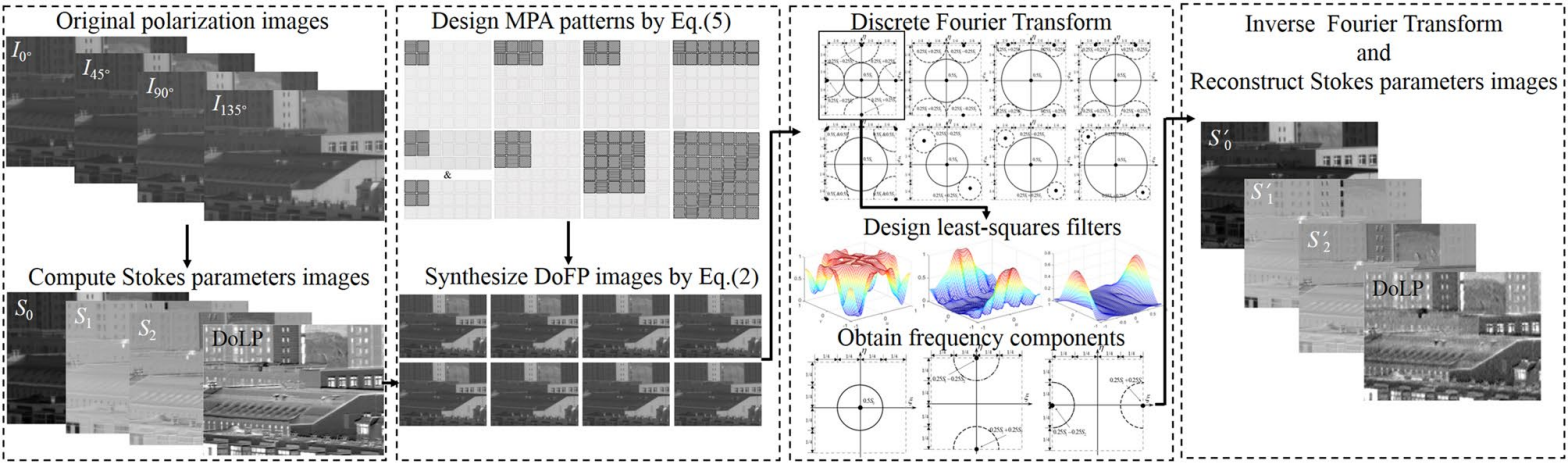
Flowchart for reconstructing the images of different Stokes parameters.

To provide a quantitative comparison of different MPA patterns, numerical simulations are also performed to evaluate the imaging quality of reconstruction. Based on Junchao Zhang’s database^15^, we simulated the reconstructed performance of Stokes parameters and DoLP through visual and quantitative measurement by comparing three new N × N diagonal MPA patterns with several mainstream MPA patterns such as 2 × 2^3^, 2 × 4^7^, 2 × 3^8^, 2 × 7^8^, 2 × 2 × 2^8^. The scheme for the reconstruction of Stokes parameters and DoLP images is described in Fig. 2, which contains four main steps: (1) by applying the true intensity images of *I*_0°_, *I*_45°_, *I*_90°_ and *I*_135°_, we can calculate the Stokes parameters and DoLP images as the true samples; (2) by combining different MPA patterns as shown in Fig. 1, we generate the synthesized DoFP images from the true Stokes parameters images of *s*_0_, *s*_1_ and *s*_2_; (3) through Discrete Fourier Transform(DFT) and frequency domain filtering, we get three frequency components, respectively; (4) by employing the Inverse Fourier Transform (IFT), we can reconstruct the Stokes parameters and DoLP images in the end.

To reconstruct the Stokes parameters and DoLP from the synthesized image, the reconstruction algorithm and filtering operation must be applied^9,16^. As shown in Fig. 1, the appropriate band-pass filters must be used to estimate the frequency components from different locations and bandwidths. Like the color filter array (CFA) designing and reconstructing process^17,18^, we want to note that the algorithms also profoundly affect the reconstruction quality. Instead of developing extremely complex reconstruction algorithm, in this paper we focus on new diagonal MPAs design and the comparison of the MPA’s inherent attribute. Considering that the practical bandwidths occupied by the frequency components are diverse for different scene targets and different MPAs, the filters based on the least-square method have more powerful abilities to be suited for various scenes and MPAs. Inspired by the reconstruction process for the Bayer CFA^19,20^, in this paper, we used the reconstruction algorithm based on the least-square filters to reconstruct Stokes parameters and DoLP. In order to estimate for the components *s*_0_, *s*_1_, and *s*_2_ by the filtering operation, the corresponding filters *h*_1_, *h*_2_, *h*_3_ were separately optimized according to the scenes and MPA patterns we mentioned. Formally, the least-square filters can be expressed by,

9 $$\begin{aligned} h^{\prime}_{x} & = \arg \min_{h} \left\| {f_{x} - \hat{f}_{x} } \right\|^{2} \\ & = \arg \min_{h} \left\| {f_{x} - {\varvec{I}}_{{\text{MPA}}} * h_{x} } \right\|^{2} , \\ \end{aligned}$$

where $h^{\prime}_{x}$ represents the obtained band-pass filters *h*_1_, *h*_2_ and *h*_3_ which are used to estimate the modulated frequency components in Fourier domain, respectively. The $f_{x}$ is the original signal obtained by the full resolution ground-truth images and $\hat{f}_{x}$ represents the estimated signal.

**Figure 3:**
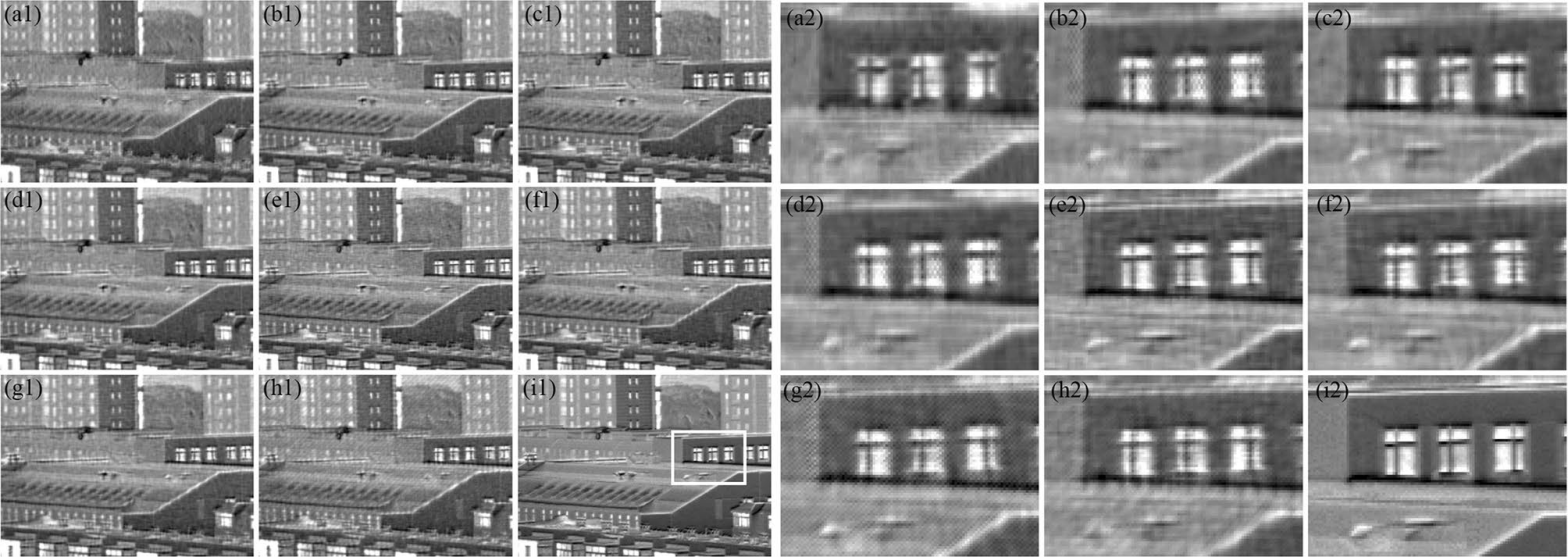
Reconstructed DoLP images of different MPA patterns on the building1 scene. The left side represents the (**a1**) 2 × 2, (**b1**) 2 × 4, (**c1**) 2 × 3, (**d1**) 2 × 7, (**e1**) 2 × 2 × 2, (**f1**) 3 × 3, (**g1**) 5 × 5, (**h1**) 7 × 7, and (**i1**) ground-truth. The right side gives the enlarged views of white rectangular regions of the reconstructed images and ground-truth image according to MPAs in the left side.

As shown in Figs. 3i1 and 4i1, two target scenes were used to evaluate the reconstructed performance. Notably, the white rectangular regions of the ground-truth image were expanded and presented in Figs. 3a2–i2 and 4a2–i2. To facilitate the objective comparisons, the peak-signal-to-noise-ratio (PSNR)^21^ was used as a quantitative evaluation criterion in this paper, which is the most common measure to assess the performance of spatial resolution enhancement. The calculation formula of PSNR is defined as:

10 $${\text{PSNR}} = 10\log_{10} ({\text{MAX}}^{2} {\text{/MSE}}).$$

**Figure 4:**
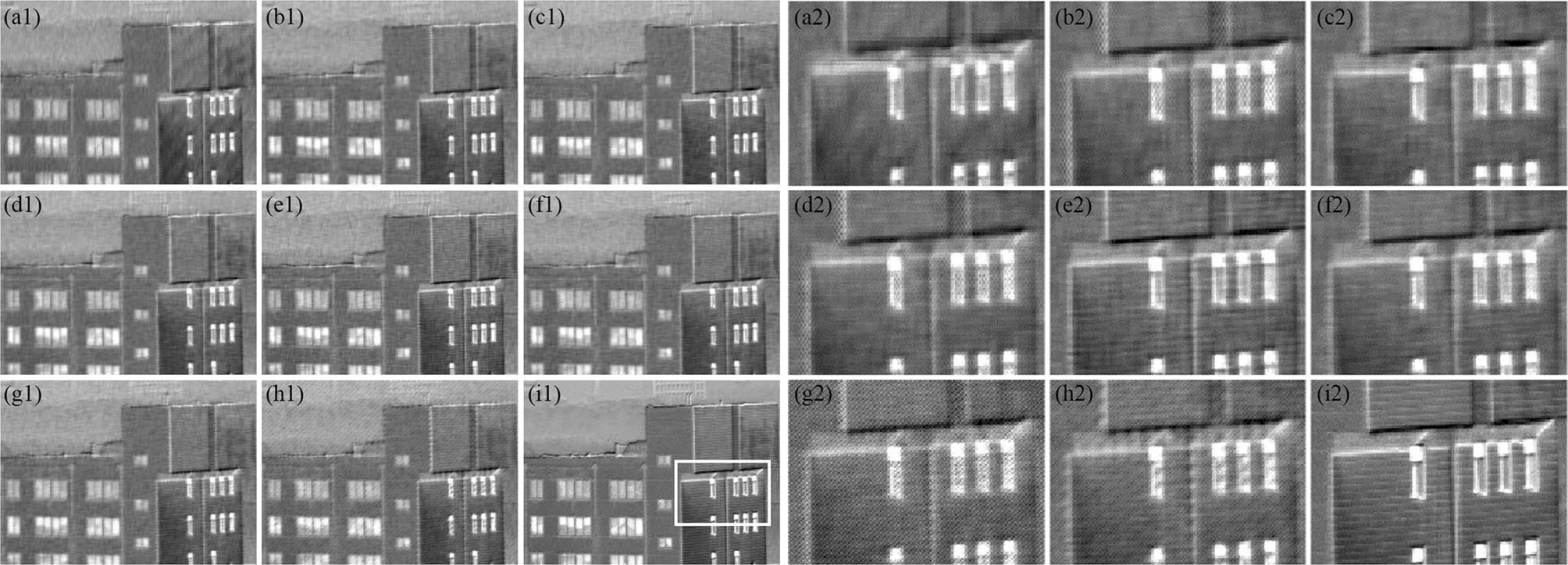
Reconstructed DoLP images of different MPA patterns on the building2 scene. The left side represents the (**a1**) 2 × 2, (**b1**) 2 × 4, (**c1**) 2 × 3, (**d1**) 2 × 7, (**e1**) 2 × 2 × 2, (**f1**) 3 × 3, (**g1**) 5 × 5, (**h1**) 7 × 7, and (**i1**) ground-truth. The right side gives the enlarged views of white rectangular regions of the reconstructed images and ground-truth image according to MPAs in the left side.

In order to give a comprehensive evaluation, the gradient magnitude similarity deviation (GMSD)^22^ was also used to measure the quality of reconstructed images, which makes use of global variation of gradient based local quality map for the overall image quality prediction

11 $${\text{GMSD}} = \sqrt {\frac{1}{N}\sum\limits_{i = 1}^{N} {\left( {{\text{GMS}}\left( i \right) - {\text{GMSM}}} \right)} } ,$$

where *N* represents the total number of pixels in the image. GMS represents the gradient magnitude similarity map and GMSM is the average of GMS. In general, the larger PSNR value is, the higher reconstruction accuracy will be, while a smaller GMSD value indicates that the reconstruction is of higher quality.

## Results and discussions

The value of PSNR and GMSD used for comparison are shown in Table 2, and the best results in each row are in boldface and underlined among the proposed MPA layouts. For the single-snapshot systems, the best results in each row are highlighted in bold, to indicate the best reconstruction performance among the single-snapshot layouts. It is obvious from Table 2 that the 2 × 2 × 2 MPA provides the best *s*_0_, *s*_1_, *s*_2_ accuracy and DoLP accuracy in all scenes, which is consistent with the theoretical analysis. Besides, the new N × N diagonal MPAs provide better Stokes parameters and DoLP reconstruction accuracy than the conventional 2 × 2 MPA in the building1 scene. In particular, the new 5 × 5 diagonal MPA provides the worst DoLP reconstruction accuracy in the building2 scene, which indicates that the frequency structure is particularly associated with the MPA pattern and the scenes. In addition, according to the PSNR and GMSD results, the 2 × 3 MPA can reconstruct the *s*_0_ with a higher accuracy than other single-snapshot layouts, and the 7 × 7 diagonal MPA comes second. Note that if we need to reconstruct Stokes parameters and DoLP images in case of single-snapshot systems, the 3 × 3 diagonal MPA provides the best *s*_1_, *s*_2_, and DoLP accuracy in all scenes, which demonstrates that our proposed new 3 × 3 diagonal MPA reconstructs state-of-the-art results. Moreover, the reconstructed DoLP images are shown in Figs. 3a1–i1 and 4a1–i1, and the enlarged views of the reconstructed images and ground-truth image are shown in Figs. 3a2–i2 and 4a2–i2 for visual comparison. As shown in Figs. 3 and 4, we can see that the reconstructed DoLP image by 2 × 2 × 2 MPA is much cleaner while preserving image details and edges, removing aliasing artifacts and reducing the zipper effect artifacts. However, in case of single-snapshot systems, it is seen that the quality of the DoLP image achieved by 3 × 3 diagonal MPA is much better. On the contrary, it can be seen clearly that the loss of image details and the generation of false grid exists in other reconstructed DoLP images. Thus, the novelty diagonal MPAs proposed in this paper can enhance the quality of the reconstruction images effectively, and our improved model has great value for DoFP polarization imaging.

**Table 2:** Reconstruction results with different MPAs.

	PSNR reconstruction results								GMSD reconstruction results (× 10^–9^)							
	2 × 2	2 × 4	2 × 3	2 × 7	2 × 2 × 2	3 × 3	5 × 5	7 × 7	2 × 2	2 × 4	2 × 3	2 × 7	2 × 2 × 2	3 × 3	5 × 5	7 × 7
**Building1**																
S_0_	42.96	47.73	**48.23**	47.98	**49.13**	47.30	47.96	**48.23**	406.80	10.41	**7.65**	10.16	**6.13**	19.88	10.89	9.41
S_1_	42.76	45.95	45.66	45.78	**49.13**	**47.15**	45.66	45.65	1193.7	285.31	326.06	256.68	**67.38**	**138.81**	153.97	272.34
S_2_	43.05	46.13	45.72	46.04	**49.10**	**46.57**	45.55	45.63	817.44	218.10	274.95	215.26	**67.99**	**157.78**	168.77	219.93
DoLP	39.03	42.87	42.26	42.44	**44.62**	**43.63**	42.56	42.43	2422.8	335.07	583.30	443.28	**148.40**	**238.67**	284.84	500.02
**Building2**																
S_0_	44.07	48.58	**49.09**	48.76	**49.94**	48.44	48.76	48.90	150.46	9.99	**6.45**	8.11	**5.54**	10.97	7.31	6.60
S_1_	44.26	47.22	47.30	46.87	**49.94**	**48.41**	41.79	45.08	755.34	181.82	295.94	256.73	**80.00**	**165.71**	187.60	246.56
S_2_	43.78	47.04	47.13	46.92	**49.60**	**47.73**	41.64	44.81	644.61	176.03	204.82	166.55	**67.29**	**143.97**	177.70	215.50
DoLP	41.76	44.07	43.98	43.78	**45.55**	**44.69**	39.45	42.17	1120.4	377.14	552.26	433.73	**199.65**	**359.87**	418.35	566.17

**Figure 5:**
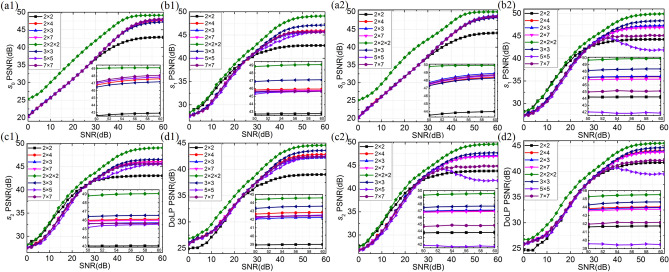
Convergent curves of the PSNR on the testing scenes with different MPA patterns. The left side on the building1 scene represents the (**a1**) PSNR value of *s*_0_, (**b1**) PSNR value of *s*_1_, (**c1**) PSNR value of *s*_2_, (**d1**) PSNR value of DoLP; the right side on the building2 scene represents the (**a2**) PSNR value of *s*_0_, (**b2**) PSNR value of *s*_1_, (**c2**) PSNR value of *s*_2_, (**d2**) PSNR value of DoLP.

Finally, to compare the capability of denoising, we first add Gaussian noise to the original images. Then the corresponding reconstructed images are generated using the different MPAs. Similarly, the PSNR is used to evaluate the reconstruction performance, and the level of the additional noise represented by the signal-to-noise ratio (SNR) is increased by every 2 dB from 0 to 60 dB. The corresponding PSNR curves are drawn in Fig. 5. Experimental results show that the value of PSNR increases as the SNR increases. For a low SNR, all the MPAs behave a similar *s*_1_, *s*_2_ and DoLP reconstruction performance, because all the filters are narrow and have a similar size. However, the 2 × 2 × 2 MPA provides the highest *s*_0_ reconstruction accuracy due to the most bandwidth. As the SNR increases, the filters are widened to include more frequency information. The 2 × 2 × 2 MPA gives the best *s*_0_, *s*_1_, *s*_2_ and DoLP reconstruction quality, and the 2 × 2 MPA exhibits the worst performance in the building1 scene. Unlike this trends, the 5 × 5 diagonal MPA exhibits the worst *s*_1_, *s*_2_ and DoLP reconstruction performance in the building2 scene. It worth noting that the relative bandwidth may behave flexible size in Fourier domain for different scenes^8^, which means the proposed MPAs may highlight their advantages and properly be applied to reconstruction, when the *s*_0_, *s*_1_ and *s*_2_ channels require less or more bandwidth. As the SNR reaches a high enough level, it is expected that the PSNR curves keep stable. In this case, a larger filter can result in a grid effect, due to the high-frequency content mixing in the extractive information. In contrast, a narrower filter leads to excessive blurring because of the information missing. All in all, the 2 × 2 × 2 MPA provides the best *s*_0_, *s*_1_, *s*_2_ and DoLP accuracy, while the 3 × 3 diagonal MPA designed by the improved model achieves state-of-the-art results in case of achieving the Stokes parameters *s*_1_, *s*_2_ and DoLP in the single-snapshot systems, which demonstrates that our improved design model can efficiently find new MPA patterns for enhancing the quality of reconstruction images.

## Conclusions

In conclusion, we propose an improved design model for constructing richer MPA patterns. This model is based on leveraging the Fourier domain and designing information carriers that yield optimal bandwidth, which can extend the 2 × N MPAs into an entirely new family of N × N diagonal MPAs. Both visual and quantitative measurements are applied to evaluate the reconstructed performance and denoising performance of *s*_0_, *s*_1_, *s*_2_ and DoLP. The experimental results show that the quality of *s*_0_, *s*_1_, *s*_2_ and DoLP reconstruction is significantly improved in terms of quantitative measures and visual quality by 2 × 2 × 2 MPA. If we need to reconstruct the Stokes parameters *s*_1_, *s*_2_ and DoLP simultaneously in case of a single-snapshot system, the 3 × 3 diagonal MPA achieves the state-of-the-art best results. This improved model widens the optimization space and can construct a number of available MPAs, which may further facilitate the practical applications of the DoFP technique.
